# Midden site selection in Dorcas gazelle: Larger is not always better

**DOI:** 10.1002/ece3.8141

**Published:** 2021-09-22

**Authors:** Alaaeldin Soultan, Abdullah Nagy, Omar Attum

**Affiliations:** ^1^ Department of Ecology Swedish University of Agricultural Sciences Uppsala Sweden; ^2^ Department of Zoology Al‐Azhar University in Cairo Cairo Egypt; ^3^ Department of Biology Indiana University New Albany Indiana USA

**Keywords:** Acacia trees, desert, Dorcas gazelle, middens

## Abstract

Dorcas gazelles are believed to use middens to mark their territories and transmit information. Given the commitment to maintaining a midden, it is believed that middens are not placed randomly. We examined how the habitat (tree height and maximum canopy) and anthropogenic disturbance (camel and human presence) influenced the selection of midden sites by Dorcas gazelles in South Sinai, Egypt. Our results showed that Dorcas gazelles did not place middens at larger trees, while favoring relatively smaller trees and shrubs where the anthropogenic disturbance and perceived hunting risk are less. Our results, in light of the previous findings, suggest that selection of midden sites is species context‐dependent behavior. In areas with less anthropogenic disturbance and hunting, Dorcas gazelles have been shown to select the largest trees of the same species as midden sites. In contract, in our study site with high anthropogenic disturbance and no protection from hunting, gazelles did not utilize the presumably optimum landmarks for midden sites. Our study showed that Dorcas gazelles instead utilized smaller trees and some shrubs that are less conspicuous and presumably less effective as advertisement sites, but safer.

## Graphical abstract

In this research, we examined how habitat and anthropogenic disturbance influenced the selection of midden sites by Dorcas gazelles. We found evidence that Dorcas gazelles avoid utilizing the presumably optimum landmarks for midden sites and instead utilized smaller trees and some shrubs that are less conspicuous and presumably less effective as advertisement sites, but safer.

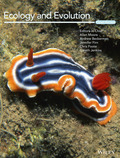

## 1 INTRODUCTION

Using olfactory signals for communication is a very common practice among mammalian species (Marneweck et al., 2017). Middens contain species‐specific pheromones (Wyatt, 2003) that reflect reliable information about the depositor such as the physical condition (Bowyer & Kitchen, 1987), the reproductive status (Miquelle, 1991), the dominance hierarchy (Miquelle, 1991), and the territorial boundaries (Attum & Mahmoud, 2012; Walther, 1978).

Communal defecation sites (henceforth referred to as “middens”) is a widespread form of scent marks (Blank et al., 2015; Wronski et al., 2006). Ungulates such as Goitered gazelle, *Gazella subgutturosa* (Blank et al., 2015); Oribi antelope, *Ourebia ourebi* (Brashares & Arcese, 1999a); Arabian gazelles, *Gazella arabica* (Wronski et al., 2013); and Dorcas gazelles, *Gazella dorcas* (Attum & Mahmoud, 2012), use middens as information centers (Marneweck et al., 2017; Wronski & Plath, 2010).

Middens allow territorial depositors to advertise their identity over a large spatial scale (Hayward & Hayward, 2010). Hence, conspecific intruders may withdraw from the territory to avoid encountering the territorial owner, which, in turn, reduces the territorial defense cost (Gosling & McKay, 1990; Hayward & Hayward, 2010). In species that have monogamous pair‐bonds such as Kirk's dik‐dik *Madoqua kirkii*, both males and females use middens for marking and defending their territories (Hendrichs, 1975). However, it is believed that female Arabian gazelles use middens for social group communication, while males use middens for establishing and defending territories (Wronski et al., 2013).

The time invested in maintaining the middens and energetic costs associated with midden deposition suggests that middens are not selected randomly (Attum et al., 2006; Gosling & Roberts, 2001; Hayward & Hayward, 2010). Middens appear to be deposited at obvious and overt sites such as elevated land or near obvious landmarks in order to maximize their detectability by the conspecifics (Attum et al., 2006; Hayward & Hayward, 2010; King & Gurnell, 2007). Umbrella thorn acacia, *Vachellia tortilis,* (henceforth referred to as “Acacia tree”) is an example of visually obvious landmarks in desert ecosystems (Attum & Mahmoud, 2012). Acacia trees are gathering places for desert ungulates who visit them for shade and food resources (Attum & Mahmoud, 2012; Halevy, 1974), which would further contribute to the value of Acacia trees as midden sites (Attum et al., 2006; Attum & Mahmoud, 2012). Previous studies showed that Arabian gazelles in Saudi Arabia and Dorcas gazelles in Egypt selected the largest and the most conspicuous trees to place their middens (Attum et al., 2006; Attum & Mahmoud, 2012).

Dorcas gazelle was once widespread and relatively common in the Qaa plain in south Sinai, Egypt (Osborn & Helmy, 1980; Saleh, 1987). In the Qaa plain, there is a high association between gazelles distribution and the presence of Acacia trees (El Alqamy, 2002; El Alqamy & Baha El Din, 2006; Saleh, 1987). In the past decade, the distribution and population size of Dorcas gazelle in the Qaa plain have declined tremendously and have largely disappeared from the vehicle‐accessible areas that consist of open plains and sandy substrates (El Alqamy & Baha El Din, 2006). This decline is believed to be a result of illegal poaching, habitat fragmentation, and human disturbance, including vehicular traffic, rock quarrying, and cutting of Acacia trees (El Alqamy & Baha El Din, 2006).

This study investigated how Dorcas gazelles select their midden sites in highly disturbed habitats and whether larger landmarks are selected for midden deposition. To this end, we examined the presence of middens in relation to tree size (tree height and maximum canopy) and anthropogenic disturbance (camel and human presence) in the Qaa plain of South Sinai, Egypt. Further, we compared the sizes (i.e., height and maximum canopy diameter) of acacia trees used and not used by humans and camels. According to the previous studies carried out in similar habitats (Attum et al., 2006; Attum & Mahmoud, 2012), we expected to find a positive correlation between the presence of middens and acacia tree size.

## 2 METHODS

### 2.1 Study area

**FIGURE 1:**
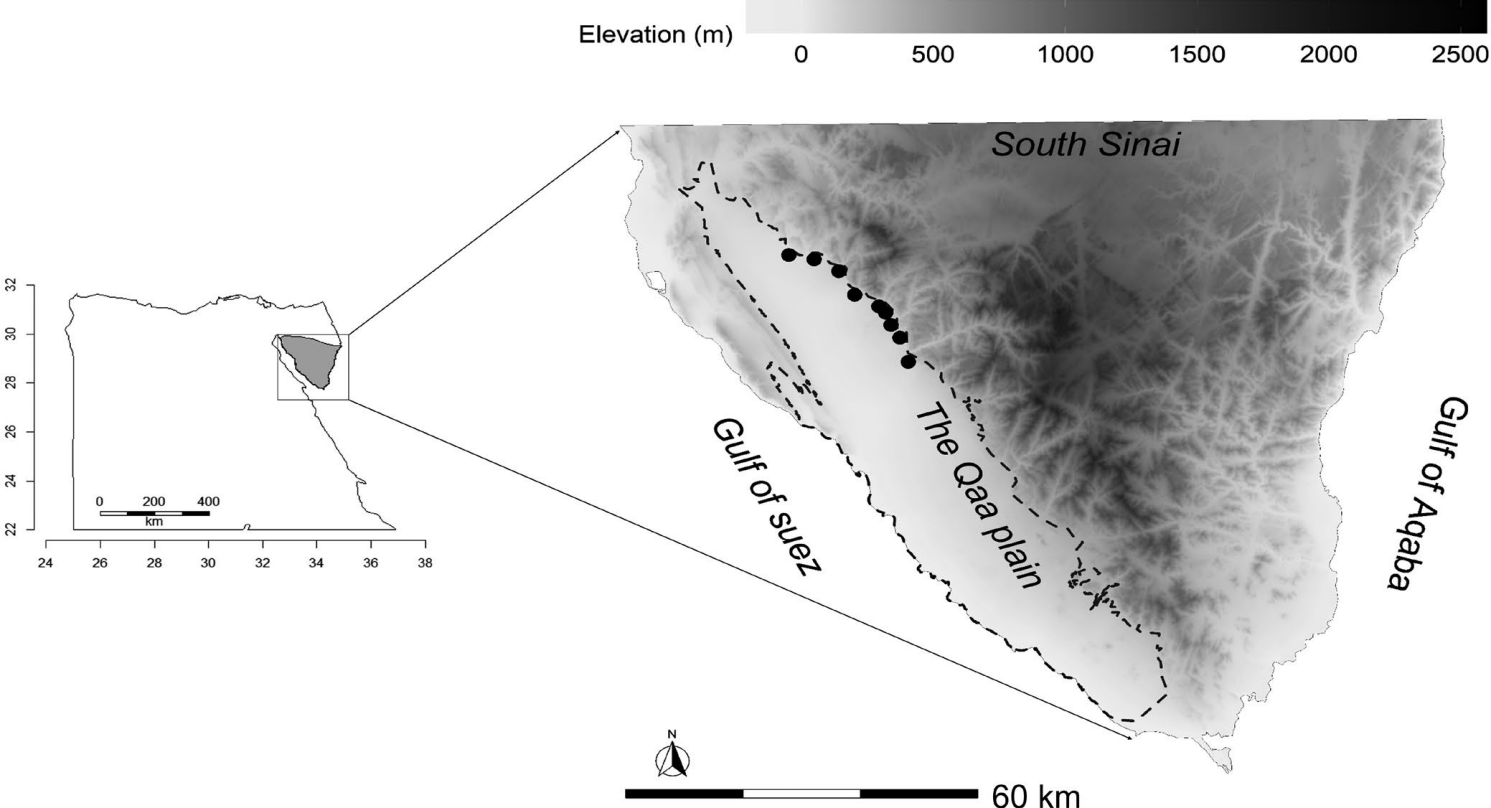
Map shows the location of the Qaa plain (dashed polygon) overplayed on the elevation map of South Sinai, Egypt. Black circle points represent the nine sites from which the midden data were collected

The Qaa plain lies at the southwestern corner of the Sinai Peninsula. The study area is bounded by mountains in the East and the Gulf of Suez in the west (Figure 1). The Qaa plain consists of an extensive gravelly and sandy plain that covers 2,000 km^2^ with scarce and patchy vegetation that follows east to west water drainage. Patches of *Vachellia tortilis* trees are clustered in the alluvial fans close to the mountain edge‐forming isolated groves (El Alqamy, 2002). Shrubs such as *Zilla spinosa*, *Panicum turgidum*, *Ochradenus buccatus*, *Asclepias sinaica*, *Retama reatem*. *Zygophyllum coccineum,* and *Capparis spinose* are common and abundant throughout the Qaa plain (El Alqamy, 2002). The Qaa plain is almost uninhabited due to the harsh environmental conditions, one of the driest regions in Egypt with annual precipitation of <30 mm (Ayyad & Ghabbour, 1986). Therefore, organized livestock (i.e., goat and sheep) grazing is rare in the Qaa plain, with free‐ranging camels the most common livestock (El Alqamy, 2002).

### 2.2 Sampling design

To identify which variables may affect the selection of midden sites, we surveyed gazelle habitat in the Qaa plain. We delineated the boundaries of the nine Acacia groves available in the study area. We randomly selected 30 points to survey among the nine patches (minimum 2 points/grove according to grove size). One out of these 30 points was excluded from our survey because it was located on the slope of a mountain. For each point, we surveyed all the trees and shrubs within a 100 m radius of the point. We then collected the following data for each point: date, time of visitation, tree and shrub species, maximum tree height (measured with a clinometer), and the maximum tree canopy diameter. If a midden was located, then we also recorded the same attributes for the nearest tree, even if it was located outside the 100 m radius. We also recorded the presence of camels, and humans within a 10 m radius from the base of a tree. A midden was defined as an accumulation of gazelle fecal pellets that covered an area of ≥50 cm^2^ (Attum et al., 2006; Attum & Mahmoud, 2012; Brashares & Arcese, 1999a, 1999b). We considered only middens with relatively fresh deposited (i.e., black or dark brown) fecal pellets (Brashares & Arcese, 1999b; El Alqamy, 2002). Camel's presence was determined through the presence of tracks or fecal pellets. Humans were considered present if there were footprints, remains of campfires, or vehicle tracks. The survey and data collection were conducted between May 27, 2012, and June 1, 2013.

### 2.3 Statistical analysis

We used a binomial generalized linear mixed model (GLMM) (Bolker et al., 2009; Zuur et al., 2009) to determine the key factors that characterize the midden sites favored by gazelles in South Sinai, Egypt. GLMM handles non‐normal data and accounts for random effects (Bolker et al., 2009). We limited the analyses to the midden records associated with only Acacia trees and excluded the other plant species. We fitted a GLMM with Acacia tree canopy diameter (continuous), camel (presence/absence), and human (presence/absence) as fixed factors and included site as a random effect to model the probability of midden occurrence using the binomial distribution with the logit link. The correlation test showed a significant positive correlation between Acacia tree height and acacia canopy diameter variables (*r* = 0.79). Therefore, Acacia tree height was not included in the GLMMs. We ran different model combinations including linear, quadratic, and interaction terms using “*glmmTMB”* function implemented in “*glmmTMB”* R package (Brooks et al., 2017) and selected the GLMM that best fit the data using Akaike's information criterion (AIC) (Pan, 2001). Further, we used MANOVA test to examine whether the size of Acacia trees (i.e., height and maximum canopy diameter) used by humans and camels was different from that of trees not used. We used human and camel presence/absence data as fixed factors, while the height and diameter of the Acacia trees were used as the dependent variables. Thus, the MANOVA was completed using the following formula:

$$\text{Acacia}\;\left[\text{height},\;\text{canopy diameter}\right]∼\text{Camel}+\text{Human}$$

We log‐transformed Acacia tree height and canopy diameter to normalize the data distribution prior to analyses. All statistical analyses were performed using R environment (R Core Team, 2016).

## 3 RESULTS

A total of 48 middens were found, 75% were found near the base of Acacia trees. Only four (8%) middens were associated with the shrub *Zilla spinosa*, one midden was associated with the shrub *Panicum turgidum*, one midden was associated with the shrub *Ochradenus buccatus*, two middens were associated with the shrub *Asclepias sinaica*, and three middens were associated with the shrub *Capparis spinosa*. One midden was not associated with vegetation and was located in an open area.

The GLMM with the best fit of the midden data had the lowest AIC value of 77.9 and included both human and camel variables as well as the quadratic term of Acacia canopy diameter (Table 1).

**TABLE 1:** A list of all models ranked according to their ΔAIC

Model rank	Model parameters	ΔAIC
1	Midden (presence/absence) ~ Human + Camel + Acacia canopy diameter + Acacia canopy diameter^2^	0.0
2	Midden (presence/absence) ~ Human + Camel * Acacia canopy diameter + Acacia canopy diameter^2^	1.7
3	Midden (presence/absence) ~ Human * Acacia canopy diameter + Camel + Acacia canopy diameter^2^	2.0
4	Midden (presence/absence) ~ Human + Camel + Acacia canopy diameter	10.5
5	Midden (presence/absence) ~ Human + Acacia canopy diameter * Camel	12.1
6	Midden (presence/absence) ~ Human * Acacia canopy diameter + Camel	12.4

**FIGURE 2:**
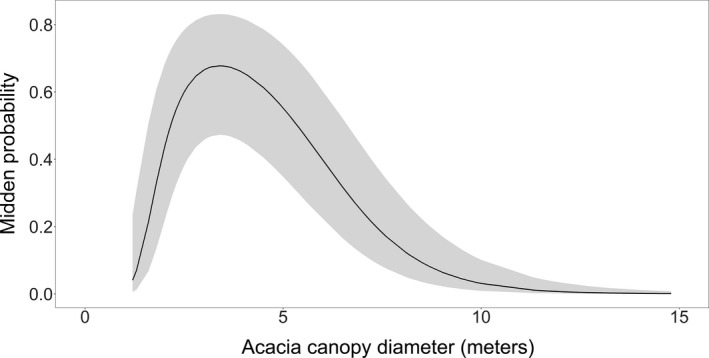
The probability of midden occurrences in response to the Acacia tree canopy diameter. The response was estimated using the generalized linear mixed model (GLMM). The black line represents the mean of the estimate, while the shaded area represents the confidence interval

This model showed significantly high explanatory (conditional *R* ^2^ = 0.79) with high predictive accuracy (*Hosmer–Lemeshow* test χ^2^ = 3.50, *df* = 3, *p* = .320). Acacia canopy diameter (quadratic term) was a significant predictor of midden occurrences (Table 2), with middens more likely to occur at Acacia trees with a canopy diameter between 3 and 6 m and less likely to occur beyond this rang (Figure 2).

**TABLE 2:** Results of the GLMM (with the lowest AIC) for the effect of Acacia tree canopy diameter and the presence of human and camel on the probability of gazelle's midden occurrences

	Estimate	*SE*	*z* value	*p*‐value
(Intercept)	−4.87	2.61	−1.87	.062
Canopy diameter	8.79	3.60	2.45	.014
I(Canopy diameter^2^)	−3.59	1.26	−2.85	.004
Human	−3.09	1.58	−1.96	.050
Camel	0.82	1.14	0.72	.473

**FIGURE 3:**
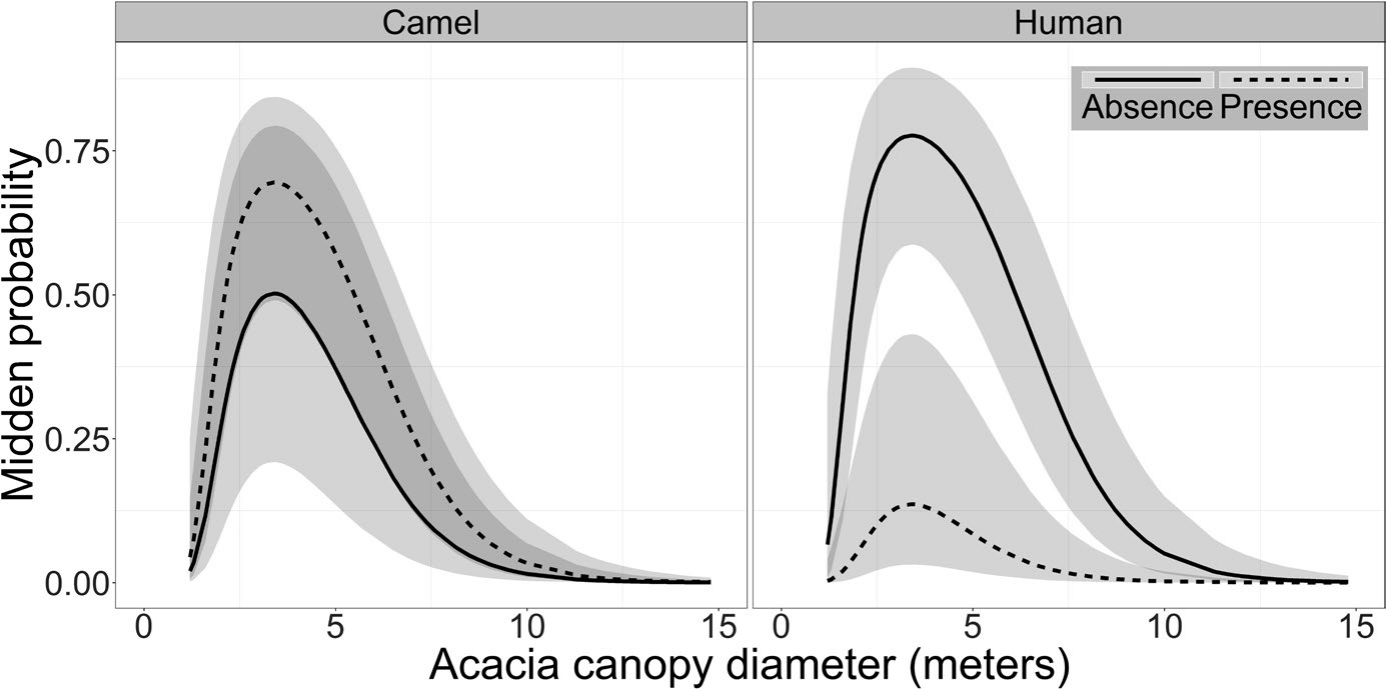
The probability of Dorcas gazelle midden occurrences in response to the Acacia tree canopy diameter conditioned to the presence of humans and camels. The response was estimated using the generalized linear mixed model (GLMM). The black line represents the mean of the estimate at the absence of camel or human, and the dashed line represents the mean estimate at the presence of camels or humans, while the shaded area represents the confidence interval

Human presence was also significant predictor of midden occurrences, with middens less likely to occur at trees visited by humans (Table 2 and Figure 3), while camel presence was not a significant predictor of midden locations (Table 2 and Figure 3).

**FIGURE 4:**
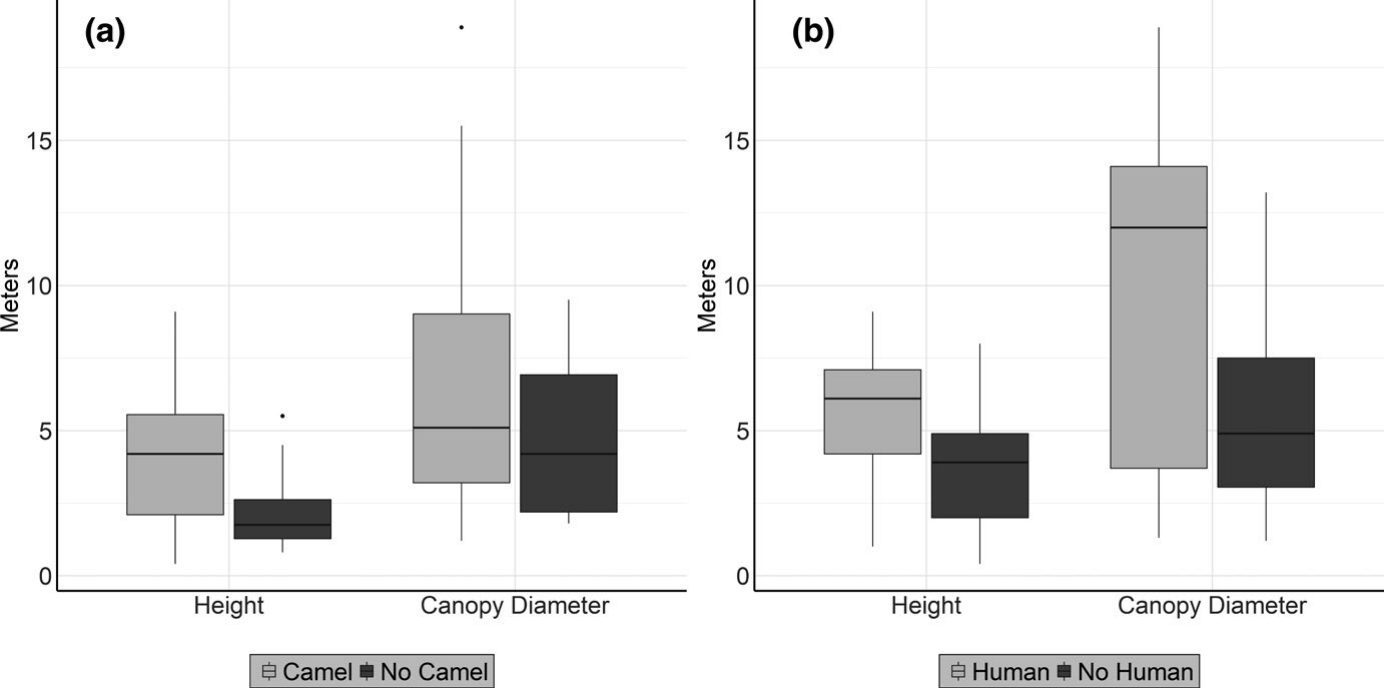
Comparison of tree size according to camel presence (a) and human presence (b). Black bars represent trees without camel (*N* = 8) and human (*N* = 67) presence. Gray bars represent trees with camel (*N* = 72) and human (*N* = 13) presence at random points

The MANOVA showed that trees used by humans (*F*_2,77_ = 3.65, *p* < .001) and camels (*F*_2,77_ = 5.53, *p* < .001) were significantly larger than trees not used. Follow‐up ANOVAs showed that trees used by camels were significantly taller (humans: *F*_1,79_ = 2.99, *p* = .087; camels: *F*_1,79_ = 6.83, *p* = .011; Figure 4a), while trees used by humans and camels had a significant wider diameter (humans: *F*_1,79_ = 6.87, *p* = .01; camels: *F*_1,79_ = 1.03, *p* = .312; Figure 4b).

## 4 DISCUSSION

Dorcas gazelles at our study site avoided placing middens at larger trees, which was in contrast to past studies that showed gazelle species select larger trees (conspicuous landmarks) to place their middens (Attum et al., 2006; Attum & Mahmoud, 2012; Salas et al., 2018). Our results also showed that humans visited larger trees as canopy diameter is correlated with tree height, with typically, taller trees having a wider canopy (Attum & Mahmoud, 2012). We believe that the gazelles do not utilize larger landmark trees for midden sites as a result of the human disturbance and hunting pressure experienced by gazelles in Sinai (El Alqamy, 2002; El Alqamy & Baha El Din, 2006). Larger trees are visited by humans to harvest seedpods for livestock, prune trees to harvest charcoal, and as shaded rest/campsites (Attum et al., 2006). In addition, hunters often visit these conspicuous landmarks to assess gazelle presence and locate tracks. Thus, gazelles may avoid these trees as midden sites because they are perceived as disturbed and dangerous habitats (Attum et al., 2006; Attum & Mahmoud, 2012; Grettenberger, 1987). Our hypothesis that gazelles avoid larger trees because they are too dangerous is further supported by past studies that occurred in a protected area (Attum & Mahmoud, 2012) or within a fenced portion of a reserve (Attum et al., 2006), with less hunting pressure, more regulated human activity, and more conservation enforcement. In contrast, the gazelle population in the Qaa plain receives no practical protection (El Alqamy & Baha El Din, 2006).

Human activities can potentially alter species behavior and affect fitness (Knight & Cole, 1995). Red deer and chamois shifted to nocturnal foraging behavior in response to human activities in New Zealand, and after controlling the human activities, they returned to diurnal foraging behavior (Knight & Cole, 1995; Pépin et al., 1996). Other species such as Bighorn sheep became more secretive and wary and spending longer time close to cover in response to human disturbance (King & Workman, 1986; Knight & Cole, 1995). Gazelles are known to respond to human disturbance similarly as they would respond to other predators (Walther, 1969). These human avoidance behaviors can be costly because it consumes the energy required for other fitness‐enhancing activities such as reproduction, foraging, and rearing offspring (Frid & Dill, 2002). For example, human disturbance influenced the reproductive behavior of Thomson gazelles, in which females may leave their breeding territories in response to hunting activities, which could lead to long‐term separation of sexes (Edington & Edington, 1986; Knight & Cole, 1995).

Although trees are the most frequent midden sites, ≈25% of the middens were distributed near small food item shrubs such as *Z. spinosa, C. spinosa,* and *O*. *baccatus*. The proportion of nontree midden sites in the Qaa plain is much higher than other gazelle populations (Attum et al., 2006; Attum & Mahmoud, 2012). We suggest these less visible shrubs could be safe gathering sites that attract gazelles in the spring/early summer for foraging and obtaining dew water in the early morning (Mallon & Kingswood, 2001; Osborn & Helmy, 1980; Ward & Saltz, 1994). We believe that the relatively high use of shrubs as midden sites in the Qaa plain further supports our hypothesis that gazelles may not be utilizing the more conspicuous larger trees as midden sites because they are too frequently disturbed by humans and perceived as dangerous. The middens near the food shrubs may still be valuable for foraging resource demarcation and communication (Brashares & Arcese, 1999a; ONO et al., 1988; Wronski et al., 2013). Similar behavior has been documented for Suni, *Neotragus moschatus* (Brashares & Arcese, 1999a), and Gerenuk, *Litocranius walleri* (Gosling, 1981).

Our results suggest that camel presence was not related to midden site selection (Figure 3). Gazelles may not perceive unattended camels as threats or as a form of disturbance. The local Bedouin community that lives near the Qaa plain releases female camels to feed on wild plants, while utilizing males for labor tasks (El Alqamy, 2002). The local community's use of camels has been practiced for hundreds of years and may have resulted in gazelles tolerating the presence of the free‐ranging camels (El Alqamy, 2002). However, gazelles and camels may still be competing for food resources, something that our study did not examine.

In conclusion, we suggest that Dorcas gazelles could be plastic in their selection of midden sites. Past studies have found in areas with less hunting and more protection, Dorcas gazelles use the largest trees for olfactory communication as these are presumably superior advertising landmarks that provide shade and food (Attum & Mahmoud, 2012). Whereas in our study site, which is heavily hunted with no protection, gazelles do not utilize the presumably optimum landmarks for midden sites and instead utilize smaller trees and some shrubs that are less conspicuous and presumably less effective as advertisement sites, but safer. Our study also suggests that although hunting has a known direct negative consequence on gazelle populations, we suggest that hunting also has secondary, indirect effect on gazelle populations by potentially altering their selection of midden sites and corresponding communication.

## CONFLICT OF INTEREST

The authors declare no competing interest.

## AUTHOR CONTRIBUTIONS

**Alaaeldin Soultan:** Conceptualization (equal); data curation (lead); formal analysis (lead); methodology (equal); visualization (equal); writing‐original draft (lead); writing‐review & editing (lead). **Abdullah Nagy:** Validation (equal); writing‐original draft (supporting); writing‐review & editing (supporting). **Omar Attum:** Conceptualization (lead); data curation (lead); formal analysis (supporting); funding acquisition (lead); investigation (lead); methodology (lead); project administration (lead); supervision (lead); writing‐original draft (supporting); writing‐review & editing (supporting).

## ETHICAL APPROVAL

Not applicable.

## Data Availability

The data that support the findings of this study are available on Dryad (https://doi.org/10.5061/dryad.05qfttf3p).

## References

[ece38141-bib-0001] Attum, O. , Eason, P. , & Wakefield, S. (2006). Conservation implications of midden selection and use in an endangered gazelle (*Gazella gazella*). Journal of Zoology, 268, 255–260. 10.1111/j.1469-7998.2005.00027.x

[ece38141-bib-0002] Attum, O. , & Mahmoud, T. (2012). Dorcas gazelle and livestock use of trees according to size in a hyper‐arid landscape. Journal of Arid Environments, 76, 49–53. 10.1016/j.jaridenv.2011.07.002

[ece38141-bib-0003] Ayyad, M. A. , & Ghabbour, S. I. (1986). Hot deserts of Egypt and Sudan. In M. Evenari , I. Noy‐Meir , & D. W. Goodall (Eds.), Ecosystems of the world, 12B, hot desert and arid shrublands (pp. 149–202). Elsevier.

[ece38141-bib-0004] Blank, D. , Ruckstuhl, K. , & Yang, W. (2015). The economics of scent marking with urine and feces in goitered gazelle (*Gazella subgutturosa*). Mammal Research, 60, 51–60. 10.1007/s13364-014-0201-1

[ece38141-bib-0005] Bolker, B. M. , Brooks, M. E. , Clark, C. J. , Geange, S. W. , Poulsen, J. R. , Stevens, M. H. H. , & White, J.‐S.‐S. (2009). Generalized linear mixed models: A practical guide for ecology and evolution. Trends in Ecology & Evolution, 24, 127–135. 10.1016/j.tree.2008.10.008 19185386

[ece38141-bib-0006] Bowyer, R. T. , & Kitchen, D. W. (1987). Significance of scent‐marking by Roosevelt elk. Journal of Mammalogy, 68, 418–423. 10.2307/1381489

[ece38141-bib-0007] Brashares, J. S. , & Arcese, P. (1999a). Scent marking in a territorial African antelope: I. The maintenance of borders between male oribi. Animal Behavior, 57, 1–10. 10.1006/anbe.1998.0941 10053066

[ece38141-bib-0008] Brashares, J. S. , & Arcese, P. (1999b). Scent marking in a territorial African antelope: II. The economics of marking with faeces. Animal Behavior, 57, 11–17. 10.1006/anbe.1998.0942 10053067

[ece38141-bib-0009] Brooks, M. E. , Kristensen, K. , van Benthem, K. J. , Magnusson, A. , Berg, C. W. , Nielsen, A. , Skaug, H. J. , Maechler, M. , & Bolker, B. M. (2017). glmmTMB balances speed and flexibility among packages for zero‐inflated generalized linear mixed modeling. The R Journal, 9, 378–400. 10.32614/RJ-2017-066

[ece38141-bib-0010] Edington, J. M. , & Edington, M. A. (1986). Ecology, recreation and tourism. Cambridge University Press.

[ece38141-bib-0011] El Alqamy, H. (2002). Developing and assessing a population monitoring program for Dorcas gazelle (*Gazella dorcas*) using distance sampling in Southern Sinai, Egypt. University of ST. Andrews.

[ece38141-bib-0012] El Alqamy, H. , & Baha El Din, S. (2006). Contemporary status and distribution of gazelle species (*Gazella dorcas* and *Gazella leptoceros*) in Egypt. Zoology in the Middle East, 39, 5–16.

[ece38141-bib-0013] Frid, A. , & Dill, L. M. (2002). Human‐caused disturbance stimuli as a form of predation risk. Conservation Ecology, 6, 11. 10.5751/ES-00404-060111

[ece38141-bib-0014] Gosling, L. M. (1981). Demarkation in a Gerenuk Territory: An economic approach. Zeitschrift Für Tierpsychologie, 56, 305–322. 10.1111/j.1439-0310.1981.tb01304.x

[ece38141-bib-0015] Gosling, L. M. , & McKay, H. V. (1990). Competitor assessment by scent matching: An experimental test. Behavioral Ecology and Sociobiology, 26, 415–420. 10.1007/BF00170899

[ece38141-bib-0016] Gosling, L. M. , & Roberts, S. C. (2001). Testing ideas about the function of scent marks in territories from spatial patterns. Animal Behavior, 62, F7–F10. 10.1006/anbe.2001.1802

[ece38141-bib-0017] Grettenberger, J. (1987). Ecology of the dorcas gazelle in northern Niger. Mammalia, 51, 527–536. 10.1515/mamm.1987.51.4.527

[ece38141-bib-0018] Halevy, G. (1974). Effects of gazelles and seed beetles (Bruchidae) on germination and establishment of acacia species. Israel Journal of Botany, 23, 120–126. 10.1080/0021213X.1974.10676842

[ece38141-bib-0019] Hayward, M. W. , & Hayward, G. J. (2010). Potential amplification of territorial advertisement markings by black‐backed jackals (*Canis mesomelas*). Behaviour, 147, 979–992. 10.1163/000579510X499434

[ece38141-bib-0020] Hendrichs, H. (1975). Changes in a population of Dikdik, Madoqua (Rhynchotragus) kirki (Günther 1880). Zeitschrift Für Tierpsychologie, 38, 55–69. 10.1111/j.1439-0310.1975.tb01992.x 1237203

[ece38141-bib-0021] King, M. M. , & Workman, G. W. (1986). Response of desert bighorn sheep to human harassment: Management implications. 51st North American Wildlife and Natural Resources Conference, 51, 74–84.

[ece38141-bib-0022] King, S. R. B. , & Gurnell, J. (2007). Scent‐marking behaviour by stallions: An assessment of function in a reintroduced population of Przewalski horses (*Equus ferus przewalskii*). Journal of Zoology, 272, 30–36. 10.1111/j.1469-7998.2006.00243.x

[ece38141-bib-0023] Knight, R. L. , & Cole, D. N. (1995). Wildlife responses to recreationists. In R. L. Knight , & K. J. Gutzwiller (Eds.), Wildlife and recreationists: Coexistence through management and research (pp. 51–69). Island Press.

[ece38141-bib-0024] Mallon, D. P. , & Kingswood, S. C. (2001). Antelopes. Part 4: North Africa, the Middle East, and Asia. Global survey and regional action plans. IUCN.

[ece38141-bib-0025] Marneweck, C. , Jürgens, A. , & Shrader, A. M. (2017). Dung odours signal sex, age, territorial and oestrous state in white rhinos. Proceedings of the Royal Society B‐Biological Sciences, 284, 20162376. 10.1098/rspb.2016.2376 PMC5247502 28077775

[ece38141-bib-0026] Miquelle, D. G. (1991). Are moose mice? The function of scent urination in moose. American Naturalist, 138, 460–477. 10.1086/285226

[ece38141-bib-0027] Ono, Y. , Doi, T. , Ikeda, H. , Baba, M. , Takeishi, M. , Izawa, M. , & Wamoto, T. I. (1988). Territoriality of Guenther's dikdik in the Omo National Park, Ethiopia. African Journal of Ecology, 26, 33–49. 10.1111/j.1365-2028.1988.tb01126.x

[ece38141-bib-0028] Osborn, D. J. , & Helmy, I. (1980). The contemporary land mammals of Egypt (including Sinai) (5th ed.). Fieldeiana Zoology, New Series. 10.5962/bhl.title.2801

[ece38141-bib-0029] Pan, W. (2001). Akaike's information criterion in generalized estimating equations. Biometrics, 57, 120–125. 10.1111/j.0006-341X.2001.00120.x 11252586

[ece38141-bib-0030] Pépin, D. , Lamerenx, F. , Chadelaud, H. , & Recarte, J. M. (1996). Human‐related disturbance risk and distance to cover affect use of montane pastures by Pyrenean chamois. Applied Animal Behaviour Science, 46, 217–228. 10.1016/0168-1591(95)00661-3

[ece38141-bib-0031] R Core Team (2016). R: A language and environment for statistical computing. R Found: Stat. Comput. http://www.R‐project.org/

[ece38141-bib-0032] Salas, M. , Manteca, X. , Abáigar, T. , Delclaux, M. , Enseñat, C. , Martínez‐Nevado, E. , Quevedo, M. , & Fernández‐Bellon, H. (2018). Using farm animal welfare protocols as a base to assess the welfare of wild animals in captivity—Case study: Dorcas gazelles (*Gazella dorcas*). Animals, 8, 111. 10.3390/ani8070111 PMC6071001 29976913

[ece38141-bib-0033] Saleh, M. A. (1987). The decline of gazelles in Egypt. Biological Conservation, 39, 83–95. 10.1016/0006-3207(87)90027-9

[ece38141-bib-0034] Walther, F. R. (1969). Flight behaviour and avoidance of predators in Thomson's gazelle (*Gazella Thomsoni* Guenther 1884). Behaviour, 34, 184–221. 10.1163/156853969X00053

[ece38141-bib-0035] Walther, F. R. (1978). Mapping the structure and the marking system of a territory of the Thornson's gazelle. African Journal of Ecology, 16, 167–176. 10.1111/j.1365-2028.1978.tb00437.x

[ece38141-bib-0036] Ward, D. , & Saltz, D. (1994). Forging at different spatial scales: Dorcas gazelles foraging for lilies in the Negev desert. Ecology, 75, 48–58. 10.2307/1939381

[ece38141-bib-0037] Wronski, T. , Apio, A. , & Plath, M. (2006). The communicatory significance of localised defecation sites in bushbuck (*Tragelaphus scriptus*). Behavioral Ecology and Sociobiology, 60, 368–378. 10.1007/s00265-006-0174-4

[ece38141-bib-0038] Wronski, T. , Apio, A. , Plath, M. , & Ziege, M. (2013). Sex difference in the communicatory significance of localized defecation sites in Arabian gazelles (*Gazella arabica*). Journal of Ethology, 31, 129–140. 10.1007/s10164-012-0357-6

[ece38141-bib-0039] Wronski, T. , & Plath, M. (2010). Characterization of the spatial distribution of latrines in reintroduced mountain gazelles: Do latrines demarcate female group home ranges? Journal of Zoology, 280, 92–101. 10.1111/j.1469-7998.2009.00643.x

[ece38141-bib-0040] Wyatt, T. D. (2003). Animals in a chemical world. In T. D. Wyatt (Ed.), Pheromones and animal behaviour (pp. 1–22). Cambridge University Press. 10.1017/CBO9780511615061.002

[ece38141-bib-0041] Zuur, A. F. , Ieno, E. N. , Walker, N. J. , Saveliev, A. A. , & Smith, G. M. (2009). GLMM and GAMM. In A. F. Zuur , E. N. Ieno , N. Walker , A. A. Saveliev , & G. M. Smith (Eds.), Mixed effects models and extensions in ecology with R (pp. 323–341). Springer. 10.1007/978-0-387-87458-6_13

